# Retrospective cohort analysis of healthcare claims in the United States characterising asthma exacerbations in paediatric patients

**DOI:** 10.1186/s40413-016-0109-0

**Published:** 2016-06-06

**Authors:** Robert Y. Suruki, Nada Boudiaf, Hector G. Ortega

**Affiliations:** UCB Biosciences Inc., 8010 Arco Corporate Drive, Raleigh, NC 27617 USA; GSK, Worldwide Epidemiology, Stockley Park West, Uxbridge, Middlesex UK; Genentech, 1 DNA Way, South San Francisco, CA 94401 USA

**Keywords:** Asthma, Exacerbations, Paediatric, Systemic corticosteroids

## Abstract

**Background:**

Asthma is the most common chronic disease in childhood and places a significant burden on public and private health systems. This retrospective cohort analysis utilised administrative healthcare claims data (US Clinformatics™ Multiplan database; compliant with the US Department of Health & Human Services Health Insurance Portability and Accountability Act) to characterise asthma exacerbations requiring intervention in a US paediatric patient population.

**Methods:**

Patients aged > 1–17 years with a recorded asthma diagnosis and receiving treatment were identified in the US Clinformatics™ Multiplan database over a 9-year period (2004–2012). Both incident and prevalent cases of asthma were included, with the most recently recorded asthma diagnosis designated as the index date. The 12-month period following the index date was analysed for asthma exacerbations, defined as an event requiring treatment with systemic corticosteroid or resulting in an asthma-related hospitalisation or emergency department visit.

**Results:**

Data from 734,114 children with asthma (41.5 % females, 58.5 % males) were analysed, of this cohort 34.4 % experienced ≥ 1 exacerbation during the follow-up period. The proportion who experienced ≥ 1 exacerbation increased from 28.9 % in 2004 to 36.3 % in 2012, based on the reported index date. Their mean annual exacerbation frequency was 1.4; 85.8 % of exacerbations were defined by systemic corticosteroids use. A consistent trend of increased exacerbation incidence in the fall and early winter was observed, in particular exacerbations defined by systemic corticosteroid use. A greater proportion of asthma-related hospitalisations were associated with younger age.

**Conclusions:**

Approximately one-third of children experienced ≥ 1 exacerbation in real-world clinical practice. A targeted treatment approach with a focus on those with a history of recurrent exacerbations is recommended to improve asthma control. This targeted approach could also minimise the frequent systemic corticosteroid exposure particularly at an early age when side effects of systemic corticosteroids are more pronounced.

## Background

Asthma has been estimated to affect up to 10 % of children in the US [1]. Furthermore, the prevalence of uncontrolled asthma in children remains high despite widespread availabilityy of effective controller medications [2–5]. Lack of adherence to maintenance therapies such as inhaled corticosteroids, may lead to poor asthma control, particularly in older children [6]. Poor asthma control is associated with increased frequency of exacerbations [7, 8] and consequent hospitalisation [2]. In view of the high prevalence of uncontrolled asthma, and considering the impact of exacerbations on patients in terms of reduced quality of life and lost school days [9], and on healthcare costs [10], improving the diagnosis and management of paediatric asthma is a high public health priority.

Asthma exacerbations are acute worsening of symptoms that often require treatment with systemic corticosteroids, visits to the emergency department (ED), or hospital admission [11]. Exacerbations are closely associated with asthma-related morbidity and mortality and are responsible for considerable healthcare costs [10]. Children account for approximately one third of all asthma-related hospitalisations [12]. Despite improvements in asthma management and advances in therapeutics, there has not been a reduction in the reported incidence of exacerbations in paediatric asthma [5]. Previously reported findings from controlled clinical trials indicate that asthma exacerbations in children [9, 13], as well as in adults [14, 15], are predictive of future exacerbations. However, data from randomised controlled trials are often not representative of real-world clinical practice.

To this end, we report the findings of a retrospective cohort study performed using a US administrative healthcare claims data to establish the frequency and type of exacerbations in 734,114 paediatric patients with asthma. Data were obtained from the Clinformatics™ DataMart Multiplan (IMPACT), a product of OptumInsight Life Sciences, Inc. (Eden Praire, MN), formerly the Integrated Healthcare Information Services database. The Clinformatics™ Multiplan database contains anonymised healthcare claims data, including information on inpatient and outpatient medical services and pharmacy dispensing claims, compliant with the Health Insurance Portability and Accountability Act (HIPAA) (US Department of Health & Human Services) on more than 90 million commercially insured patients, primarily from the UnitedHealth Group [16].

In this study, we analysed the annual frequency of paediatric exacerbations in real-world clinical practice and examined any association of age group or gender with exacerbation frequency. Additionally, the longitudinal nature of the dataset was utilised to investigate exacerbation recurrence and seasonal trends in exacerbations.

## Methods

### Study design

Patients aged > 1–17 years with asthma were identified in the Clinformatics™ Multiplan administrative healthcare claims database in the US by the identification of an outpatient or inpatient claim coded with asthma (ICD9 493.xx) during the period January 1, 2004 and ending on December 31, 2013; the index date was defined as the date of the most recently recorded asthma medical code during the study period that met all inclusion and exclusion criteria. The study period (2004–2013), included a 10-year observation period, and data were assessed in a *post-hoc* analysis, since the original study period was up to 3 years. Patients identified in the database were required to have at least one recorded asthma medication (e.g. short-acting beta agonist, inhaled corticosteroid [ICS], ICS plus long-acting beta agonist [LABA], leukotriene receptor antagonist [LTRA] or omalizumab) dispensing within +/–3 months of asthma diagnosis, and at least 12 months of data coverage subsequent to the asthma index date.

Patients were excluded from the cohort if their records indicated a history of cystic fibrosis or chronic obstructive pulmonary disease.

### Study endpoints

The event of interest in this retrospective cohort study was asthma exacerbations, defined as treatment with systemic corticosteroids administered both orally and via injection, asthma-related ED visits, or asthma-related hospitalisations. Asthma related ED visits and hospital admissions were determined by the presence of an asthma primary diagnosis and/or discharge diagnosis.

### Statistical analysis

Data were extracted from the Clinformatics™ Multiplan database and maintained and analysed using SAS v9.2 software (SAS Institute Inc., Cary, NC, USA). All patient-level data were anonymised before being made available for analysis in this study and US data collection was compliant with HIPAA. Therefore, use of these data in clinical research is exempt from Institutional Review Board review.

ICD-9 codes were used to select patients with asthma and healthcare utilisation associated with asthma. The subsequent 12-month period was queried for recorded systemic corticosteroid tablet treatment of 3 or more days (systemic corticosteroids administered via injection were not required to have a minimum duration). ED visits and hospitalisations with an associated asthma-related diagnosis code were also captured during this period. Systemic corticosteroid dispensing occurring less than 7 days apart was considered a single event.

We assessed the overall incidence and type of exacerbation, and the impact of patient demographic factors (age and gender) on exacerbation frequency. Exacerbation recurrence and the relationship between use of controller medications (e.g. LTRA, ICS, ICS/LABA, systemic corticosteroid, omalizumab, theophylline) and exacerbation frequency were also assessed. A sensitivity analysis was performed to evaluate the effect of exclusion of patients in the cohort whose claims history indicated comorbid chronic inflammatory disease (Table 1). The presence of a comorbid chronic inflammatory disease that may have required the use of systemic corticosteroids at any time during the patient’s medical history was defined as having an ICD-9 code as listed in Table 1.

**Table 1:** Chronic inflammatory diseases with ICD-9 codes included in the sensitivity analysis

Chronic inflammatory disease	ICD9 code
Systemic lupus erythematosus	(710.0)
Juvenile ankylosing spondylitis	(720.0)
Scleroderma	(710.1)
Inflammatory bowel disease	(555.x)
Vasculitis	(447.6)
Multiple sclerosis	(340)
Psoriasis	(696.x)
Juvenile rheumatoid arthritis	(714.3, 714.30, 714.31, 714.32, 714.33)
Juvenile dermatomyositis	(710.3)
Idiopathic thrombocytopenic purpura	(287.31)
Necrotizing enterocolitis	(777.5)
Kawasaki disease	(446.)
Celiac disease	(579.0)
Sickle cell anaemia	(282.6x)

Data were interpreted descriptively. Based on previous analyses of the Clinformatics™ Multiplan databases, it was determined that the number of paediatric asthma patients likely to be present in the database would be sufficient to achieve the aims of the study.

## Results

### Overview of patient population and exacerbation data

Data for 734,114 paediatric patients with asthma who met the criteria for data inclusion were analysed (Table 2). The overall study population comprised more males than females (58.5 % vs. 41.5 %); age ranged from > 1 to 17 years.

**Table 2:** Demographic and exacerbation data derived from the study cohort

Characteristics	No exacerbations	≥1 exacerbation		Total			
	*n*	Column %	*n*	Column %	Row %	*n*	Column %
Total	481,822	100.0	252,292	100.0	34.4	734,114	100.0
Gender							
Male	276,033	57.3	153,216	60.7	35.7	429,249	58.5
Female	205,789	42.7	99,076	39.3	32.5	304,865	41.5
Age at date of asthma diagnosis							
≤ 1 year	25,495	5.3	20,437	8.1	44.5	45,932	6.3
> 1–2 years	24,329	5.0	20,226	8.0	45.4	44,555	6.1
> 2–3 years	25,722	5.3	19,414	7.7	43.0	45,136	6.1
4–12 years	262,219	54.4	133,291	52.8	33.7	395,510	53.9
13–17 years	144,057	29.9	58,924	23.4	29.0	202,981	27.6
Asthma index date^a^							
2004	41,964	10.3	17,066	8.9	28.9	59,030	9.8
2005	45,312	11.1	18,947	9.9	29.5	64,259	10.7
2006	51,128	12.6	21,546	11.2	29.6	72,674	12.1
2007	45587	11.2	20,957	10.9	31.5	66,544	11.1
2008	38,942	9.6	18,952	9.9	32.7	57,894	9.7
2009	40,484	10.0	20,025	10.4	33.1	60,509	10.1
2010	38,010	9.4	19,044	9.9	33.4	57,054	9.5
2011	42,088	10.4	19,975	10.4	32.2	62,063	10.4
2012	62,761	15.4	35,703	18.6	36.3	98,464	16.5

^a^Values are for patients 4–17 years at asthma index date

**Fig. 1:**
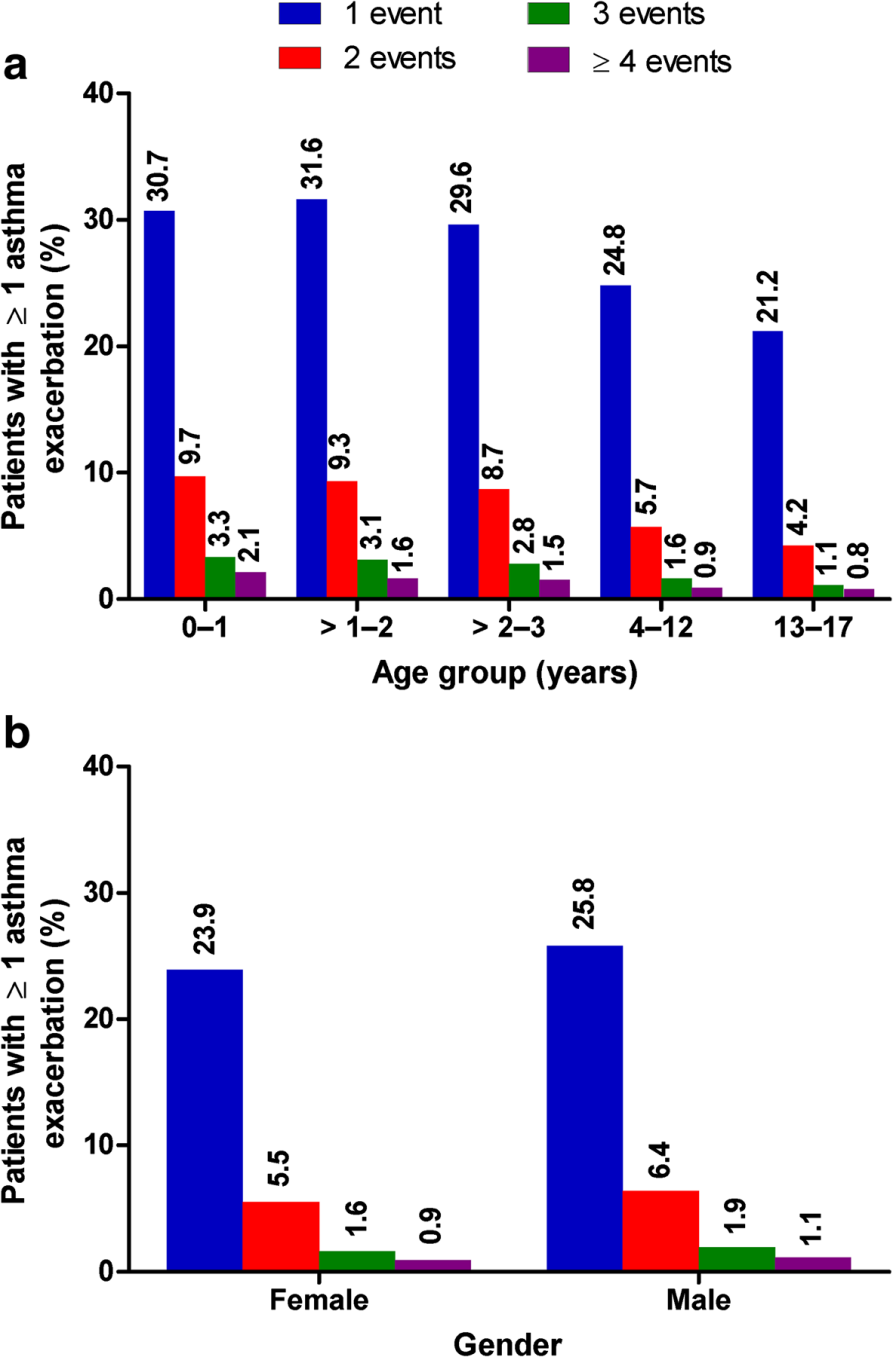
Exacerbation frequency during the 12-month follow-up period by **a** age group and **b** gender

In the study cohort, 34.4 % experienced ≥ 1 exacerbation during the 12-month follow-up period (males 60.7 %; females 39.3 %). Increasing age was associated with decreased frequency of exacerbations and reduced incidence of exacerbation recurrence (Table 3). The overall mean exacerbation frequency for patients experiencing ≥ 1 exacerbation during the follow-up period was 1.4 events per person (standard deviation = 0.9), with a higher incidence of recurrence observed in the younger age groups compared to the older children and adolescents (Table 2 and Fig. 1a). The proportion of patients with at least one exacerbation during the 12-month follow-up period increased from 2004 to 2012 (Table 2). No difference in frequency of recurrent exacerbations by gender was observed (Fig. 1b).

**Table 3:** Asthma exacerbation frequency among the study cohort paediatric asthma patients, by gender and age group

	Number of exacerbations during 12 month follow-up period					Total
	0	1	2	3	≥4	
	*n* (%)	*n* (%)	*n* (%)	*n* (%)	*n* (%)	*N*
Overall	481,822 (65.6)	189,457 (25.8)	43,592 (5.9)	12,278 (1.7)	6,965 (1.0)	734,114
Gender						
Female	205,789 (67.5)	75,394 (24.7)	16,517 (5.4)	4,530 (1.5)	2,635 (0.9)	304,865
Male	276,033 (64.3)	114,063 (26.6)	27,075 (6.3)	7,748 (1.8)	4,330 (1.0)	429,249
Age groups						
0–1 years	25,495 (55.5)	14,078 (30.7)	4,218 (9.2)	1,359 (3.0)	782 (1.7)	45,932
> 1–2 years	24,329 (54.6)	14,176 (31.8)	4,084 (9.2)	1,282 (2.9)	684 (1.5)	44,555
> 2–3 years	25,722 (57.0)	13,904 (30.8)	3,742 (8.3)	1,138 (2.5)	630 (1.4)	45,136
4–12 years	262,219 (66.3)	101,711 (25.7)	22,297 (5.6)	6,077 (1.5)	3,206 (0.8)	395,510
13–17 years	144,057 (71.0)	45,588 (22.5)	9,251 (4.6)	2,422 (1.2)	1,663 (0.8)	202,981

A sensitivity analysis of the study population excluding patients with any history of comorbid chronic inflammatory disease showed that exclusion of these patients (*n* = 13,036) did not affect the overall findings, with the overall exacerbation frequency being near identical (1.4 vs. 1.4).

### Exacerbation type

Summary statistics on exacerbation type data were generated (Table 4). Systemic corticosteroid-defined exacerbations (i.e., outpatient treatment) accounted for nearly 85.8 % of recorded events. Among the observed exacerbations, there were a greater proportion of ED visit-defined exacerbations (not precluding the use of systemic corticosteroids) compared with hospitalisation-defined exacerbations (11.8 % versus 2.4 %, respectively). Although a greater proportion of asthma-related hospitalisations were associated with younger age, there was no variation by gender.

**Table 4:** Asthma patients with ≥ 1 exacerbation (*n* = 252,292) by first exacerbation type recorded during the study period

Specified type	*n* (%)
Hospitalisation	6,080 (2.4)
Emergency department visit	29,713 (11.8)
Systemic corticosteroid^a^	216,499 (85.8)

^a^Includes corticosteroid injection

### Seasonal distribution of exacerbations

**Fig. 2:**
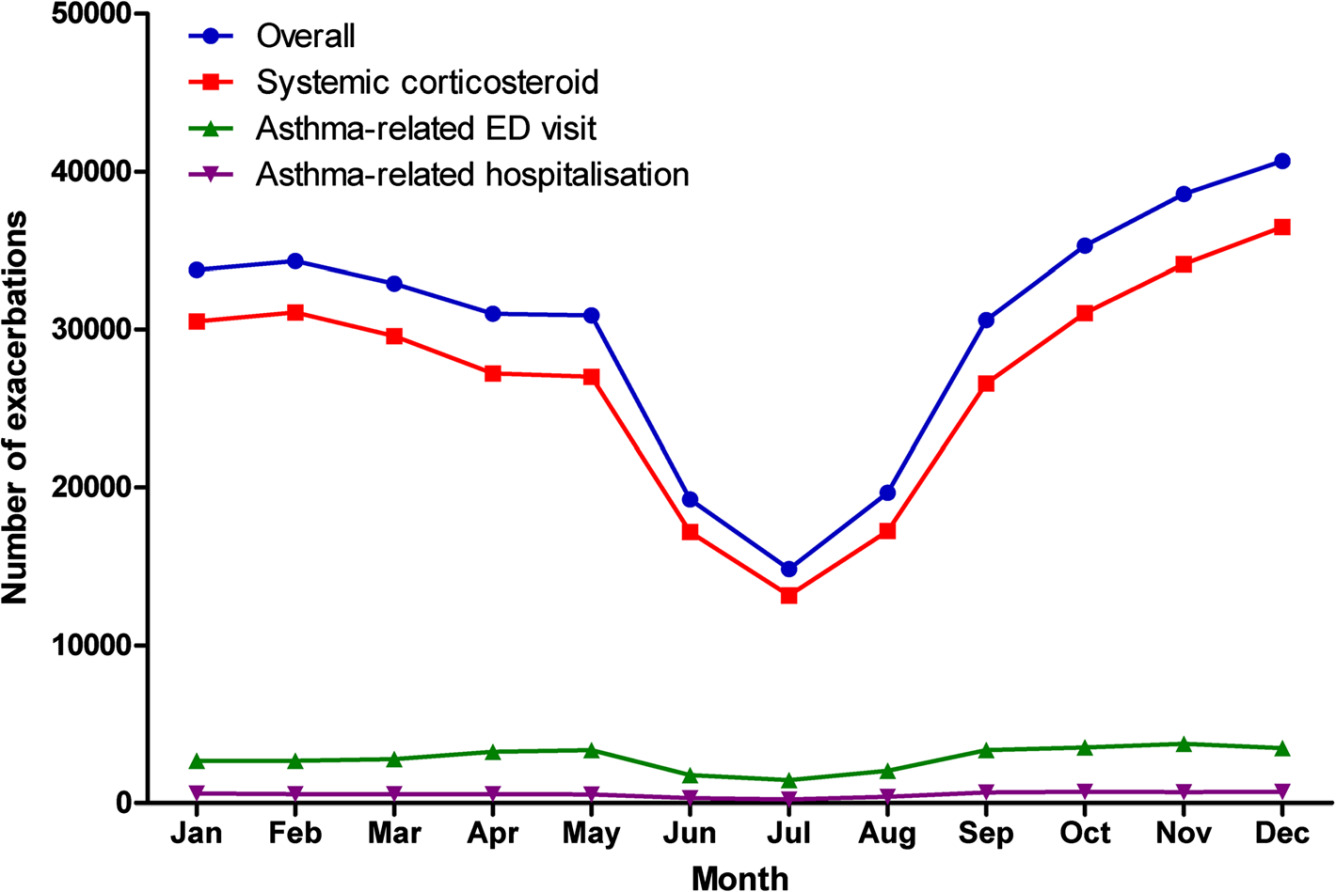
Asthma exacerbations in paediatric patients with asthma by month, overall and by exacerbation type

Seasonal variation in exacerbation frequency was analysed for all exacerbations and by type of exacerbations (Fig. 2). A trend of seasonality was observed where the exacerbation frequency was the highest in the fall and early winter and lowest during the month of July. The trend was most pronounced for systemic corticosteroid-defined exacerbations, though a similar trend can be seen for ED visit-defined and hospital-defined exacerbations.

### Association between controller medication use and exacerbation frequency

**Fig. 3:**
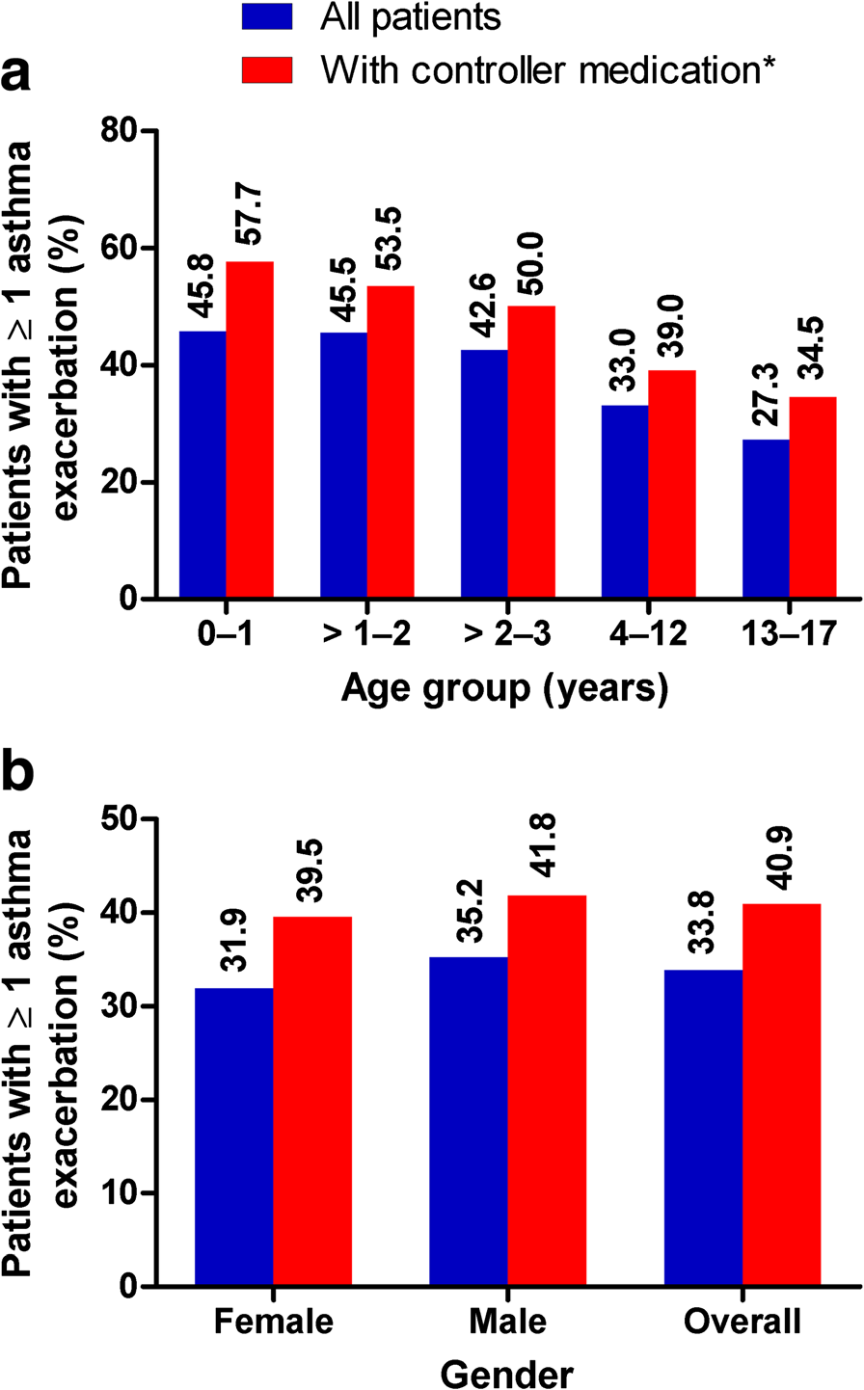
Proportion of patients with ≥ 1 exacerbation during the 12-month follow-up by **a** age and **b** gender. *The subset of asthma patients on controller medications was defined as follows: patients who had received an asthma controller medication (LTRA, ICS, ICS/LABA, OCS or injectable steroid, omalizumab, theophylline) within +/-3 months of the index date

Exacerbation frequency among patients in the cohort was analysed, overall and by age and gender, according to whether or not there was evidence that the patient had been prescribed asthma controller medication at the asthma index date (Fig. 3). A trend whereby patients prescribed controller medication at baseline were more likely to have had ≥ 1 exacerbation during the 12-month follow-up period was identified; no effect of gender was seen. Although there was no significant variation in exacerbation rates between groups defined by asthma controller use at baseline or gender, a trend towards a higher rate of exacerbations with younger age was observed (Table 5). There were very few reports of exacerbations with omalizumab; 18 males with a mean (SD) exacerbation frequency of 1.50 (0.86) and 18 females with 1.83 (1.47) exacerbations. As there were so few cases, these data have not been included in Table 5.

**Table 5:** Mean annual exacerbation rate by asthma therapy

Age, years	ICS^a^		ICS/LABA		LTRA		OCS	
	*n*	Mean exacerbation rate (SD)	*n*	Mean exacerbation rate (SD)	*n*	Mean exacerbation rate (SD)	*n*	Mean exacerbation rate (SD)
Females								
0 -1	2017	1.50 (0.89)	5	1.60 (0.89)	490	1.67 (1.07)	3502	1.42 (0.83)
>1 – 2	2036	1.49 (0.89)	10	2.0 (2.0)	1060	1.59 (1.08)	3709	1.37 (0.74)
>2 – 3	2084	1.46 (0.86)	17	1.35 (0.79)	1440	1.56 (1.0)	3289	1.34 (0.77)
4 – 12	11321	1.36 (0.83)	2034	1.42 (0.93)	12209	1.44 (0.89)	20719	1.29 (0.81)
13 – 17	3693	1.35 (0.93)	3227	1.42 (0.93)	5915	1.45 (1.06)	11070	1.39 (1.01)
Overall 0-17	21151	1.40 (0.86)	5293	1.42 (0.93)	21114	1.46 (0.96)	42289	1.34 (0.86)
Males								
0–1	4552	1.60 (1.02)	6	2.0 (0.63)	1232	1.68 (1.09)	6112	1.47 (0.89)
>1–2	3950	1.52 (0.94)	22	1.91 (1.06)	2042	1.65 (1.08)	5803	1.42 (0.81)
>2–3	3630	1.48 (0.93)	40	1.93 (1.46)	2446	1.56 (1.02)	5114	1.40 (0.89)
4–12	19133	1.39 (0.84)	3743	1.42 (0.84)	21698	1.45 (0.92)	32895	1.32 (0.84)
13–17	4208	1.32 (0.85)	3777	1.37 (0.91)	21698	1.45 (0.92)	13397	1.35 (0.92)
Overall 0–17	35473	1.43 (0.89)	7588	1.40 (0.88)	34104	1.45 (1.06)	42289	1.34 (0.86)

^a^includes nebulized medications

*ICS* inhaled corticosteroid, *LABA* long-acting beta agonist, *LTRA* leukotriene receptor antagonist, *OCS* oral corticosteroid

## Discussion

This study used a US population-based healthcare claims database to investigate paediatric exacerbation frequency. These findings suggest that approximately one-third of paediatric asthma patients (<1–17 years of age) will experience an exacerbation within a 12-month period, with a smaller subset of patients (<9 %) experiencing ≥ 2 exacerbations. Despite being a small proportion of the overall population, this patient group with evidence of more severe asthma could have a disproportionate impact on overall healthcare burden.

Diagnosis of asthma in children aged ≤ 5 years, and especially in infants, is a particular challenge [17]. Symptoms typically characteristic of asthma, such as wheezing and cough, are common in this age group and not necessarily indicative of asthma, and viral infections such as bronchiolitis can confound asthma diagnosis; furthermore, diagnostic tests such as spirometry are often not possible because of the requirement for the patient to perform voluntary breathing manoeuvres in a reproducible manner [11]. As only patients with a recorded asthma diagnosis and at least one asthma medication were included in the cohort, it is possible that asthma prevalence in this age group may be misclassified (e.g. under-reported).

It should be noted that, because of limitations of the available data resource, the definition of asthma exacerbations used in this retrospective cohort study differed from the American Thoracic Society and European Respiratory Society consensus definition [18]. While we anticipate that most severe exacerbations (requiring hospitalisation and/or ED visits) be treated with systemic corticosteroids and be recorded in the claims database, moderate or mild exacerbations may not have been fully captured, specifically those that did not require treatment with a systemic corticosteroid. Nearly 86 % of recorded exacerbations were identified through dispensed systemic corticosteroid and approximately 14 % required hospitalisation or ED visits (not precluding the use of systemic corticosteroids). It is unclear why we observed an increase in exacerbation frequency between 2004 and 2012. The US Centers for Disease Control and Prevention has published data showing that hospitalisation frequency rates remained unchanged from 2001 to 2009, whereas the prevalence of asthma increased [19]. These findings suggest that the increased exacerbation frequency observed in our study may be attributable to an increase in oral corticosteroid-defined exacerbations, rather than an increase in hospitalisations. Another possible explanation, of which further exploration may be warranted, is that the increase in exacerbation frequency may result from incremental improvements in data completeness (i.e. better recording of hospitalisations and ED visits and/or improvements in medication dispensing/prescribing data).

Oral corticosteroids are recommended for the first-line treatment of acute exacerbations in children [11]. However, the adverse impact of the chronic use of systemic corticosteroids was assessed recently in a real-world study of patients with severe asthma. Results from this assessment showed that there was a dose-dependent increase in the risk of developing infections, and/or gastrointestinal and cardiovascular complications in patients aged > 12 years who received daily systemic corticosteroids [20]. Although in the present analysis inclusion of systemic corticosteroid prescription data enabled exacerbations that did not require hospitalisation or ED visits to be captured. Systemic corticosteroid use for reasons other than asthma exacerbation could not be ruled out, and therefore some spurious exacerbations may have been recorded. To account for this possibility, a sensitivity analysis was performed evaluating a subset of patients without comorbid chronic inflammatory conditions (for which patients may have been prescribed systemic corticosteroid without having experienced an asthma exacerbation). This sensitivity analysis did not identify any decrease in the frequency of recorded asthma exacerbations.

A seasonal pattern of paediatric asthma exacerbations is well established [21–24]. In particular, a peak in asthma exacerbations and asthma-related hospitalisations in September has been widely observed in children in the Northern Hemisphere [22–25]. Furthermore, a link between asthma exacerbations and falling temperatures has also been observed [26]. Our data reflect these previously reported findings, showing a marked increase in exacerbations from August to September. This autumnal peak coincides with children returning to school and having a greater exposure to viral infections [24]. There is considerable evidence to support a causal link between viral respiratory tract infections and asthma exacerbations in children, with respiratory viruses being detected in over 80 % of paediatric patients who experience asthma exacerbations [27–30].

When the current analysis was restricted to the subset of patients using controller medications at the asthma index date, the proportion of patients experiencing an exacerbation increased. Consistent with previously reported findings, this suggests that exacerbations occur more frequently in patients who have evidence of more severe asthma and are, therefore, more likely to be prescribed ICS, in accordance with asthma management guidelines and medication labelling [11, 31]. By extension, this suggests that if stricter selection criteria (e.g. excluding patients with mild asthma, who represent a large proportion of the overall asthma population) had been used, the reported exacerbation frequency may have been greater than our results suggest. This observation should be interpreted in light of the fact that patients were not stratified by baseline asthma severity, nor does the methodology used take into account adherence or compliance to medication or comparison with patients who were undertreated.

Previously, managed care data from US healthcare claims databases have been used to investigate exacerbation frequency and associated factors in patients of all ages [5, 32]. Results from a study of adults with asthma show that the risk of exacerbation does not appear to change over time, even with high-intensity treatment, but the occurrence of previous exacerbations does increase the risk of subsequent events [33]. In this study, we made use of these data to specifically examine paediatric exacerbation frequency using a retrospective cohort approach. Our findings indicate that the occurrence of exacerbations among children diagnosed with asthma is as high in real-world clinical practice as has been previously suggested by the smaller-scale studies mentioned above. Recurrence of exacerbations requiring treatment with systemic corticosteroid or more intensive intervention is observed in < 10 % of the paediatric asthma population, and the healthcare burden associated with childhood asthma is heightened during the fall season. Although treatment of asthma has improved since 2004 our results suggest a trend towards an increase in exacerbations as the proportion of patients with at least one event, identified in the index date, increased over the course of the study (28.9 % in 2004 to 36.3 % in 2012).

The current findings are relevant as, although there are some results in the published literature describing exacerbation frequency in adults with asthma [33], there are limited data in paediatric patients with asthma identified in a large healthcare claims database, including a breakdown of exacerbation frequency by type. Moreover, the large sample size utilised to quantify the frequency of asthma exacerbations in this paediatric patient population can be considered a strength of the current study.

## Conclusions

These findings highlight the potential unmet need in approximately one-third of paediatric patients with asthma who experienced an exacerbation. This is particularly important because paediatric patients are more susceptible to adverse effects associated with the chronic use of systemic corticosteroids. We propose that a more targeted treatment approach with particular focus on those who have a history of recurrent exacerbations, together with improved treatment engagement of patients and their parents or guardians, in combination with the prescription of effective controller medications, could result in meaningful improvements in asthma control in children.

## Abbreviations

ED: emergency department
HIPAA: Health Insurance Portability and Accountability Act
ICS: inhaled corticosteroids
LABA: long-acting beta agonist
LTRA: leukotriene receptor antagonist
OCS: oral corticosteroid
